# Salicylate metabolism in Populus

**DOI:** 10.1186/1753-6561-5-S7-I9

**Published:** 2011-09-13

**Authors:** Chung Jui Tsai, Wenbing Guo, Benjamin Babst, Batbayar Nyamdari, Yinan Yuan, Raja Payyavula, Han-Yi Chen, Xue Liangjiao, Kate Tay, Vanessa Michelizzi, Scott Harding

**Affiliations:** 1 University of Georgia, USA; 2 Michigan Technological University, USA

## 

Phenolic metabolites that contain salicylic acid (SA)-like moieties are major non-structural constituents in *Populus* leaves, shoots and roots. These so-called phenolic glycosides (PGs) are taxonomically limited to the Salicaceae, where they are known to mitigate insect and animal herbivory. SA itself is an important signaling molecule in plant defense and abiotic stress responses, and is derived from the isochorismate or phenylpropanoid pathways, depending on the species [1,2]. Hyper-accumulation of SA reduces growth in *Arabidopsis*[3], while high levels of PG have been associated with reduced growth in *Populus*[4]. Although the common PGs, salicin, salicortin, and their derivatives contain a salicyl moiety, a direct metabolic relationship between PG and SA in *Populus* has not been shown. We have therefore been using stable isotope tracers, cell culture feeding, functional genomics and transgenic perturbation to probe the relationship between SA, PG and growth in *Populus*.

Stable isotope incorporation confirmed a hydroxycinnamate-benzoate origin of the salicyl moiety of PGs, and provided the first empirical evidence that the bioactive hydroxycyclohexenone moiety of salicortin is also a phenylpropanoid derivative [5]. Therefore it is unlikely that PG biosynthesis involves the salicylic acid pathway. To further address any possible involvement of the isochorismate pathway in PG biosynthesis, isochorismate synthase (ICS) was characterized. *ICS* is present as a single-gene in most sequenced plant genomes, but is duplicated in *Arabidopsis*. In *Arabidopsis*, chorismate-derived SA is the obligatory route for defense signaling, mediated by *AtICS1* in response to various biotic and abiotic cues. In contrast, the single-copy *Populus ICS* is not stress-inducible, and is involved in biosynthesis of phylloquinone for photosynthetic electron transport [2]. We found no evidence of *ICS* involvement in SA or PG biosynthesis in transgenic *Populus*, pointing to lineage-specific evolution of the ICS-derived SA pathway in *Arabidopsis*.

We transformed *Populus* with the bacterial genes for the biosynthesis and degradation of SA for a more in-depth investigation of the possibility of an SA interaction with PG regulation. SA-hyperaccumulating and SA-deficient lines were generated by expressing a salicylate synthase and a salicylate hydroxylase, respectively. SA was converted to SA-glycoside (SAG) and gentisic acid-glucoside (GAG) in the hyperaccumulating lines. SAG and GAG were very low in wild-type and were not detected in the SA-deficient lines. Despite these clear differences, no changes in PG levels were detected. These results argue against SA as potential precursor of PGs. Survival and establishment of rooted cuttings were negatively affected by SA-hyperaccumulation. Once established, however, growth rates were similar among plant lines, in sharp contrast to SA-over-producing *Arabidopsis*. SA-hyperaccumulating lines exhibited altered thermal tolerance, based on electrolyte leakage assays and metabolite profiling. Together, our results provide support for a phenylpropanoid origin of PGs, and argue against the involvement of SA in PG biosynthesis. The data is consistent with complementary roles for SA and PG in *Populus* fitness.

A number of other experiments were conducted to investigate the regulation of PG homeostasis in *Populus*. Administration of a putative PG precursor salicyl alcohol to cell cultures yielded the glucoside, salicin. Salicyl alcohol-feeding also altered the partitioning of carbon into condensed tannin (CT) biosynthesis, another quantitatively important phenylpropanoid product [6]. Salicyl alcohol-feeding induced expression of several glycosyltransferases (GT1), as well as genes associated with sucrose catabolism, glycolysis and the Krebs cycle, presumably to accommodate the increased glycosylation. Conversely, transcript levels of most of the flavonoid pathway genes were reduced, consistent with reduced CT synthesis. Given that glycosylation was the only PG biosynthetic step occurring in the fed-cells, the results suggest that glycosylation of phenolic products can play a role in regulating the tradeoff between competing chemical defenses, *e.g.*, PGs and CTs.

Correlation analysis was used to identify PG-coregulated genes from a number of inductive treatments. One such candidate, *SUT4* encoding a tonoplast sucrose transporter, was of particular interest because of the reported salicin transport activity of its *Arabidopsis* ortholog. RNAi-silencing of *SUT4* altered sucrose distribution between source and sink organs, and reduced PG accrual by 20-30%, accompanied by altered biomass partitioning [7]. Chemical and molecular analyses revealed a complex network linking PG accrual and carbohydrate homeostasis.

PG-coregulated GT1 members were also targeted for characterization, due to the prominent role of glycosylation in PG biosynthesis. We identified a novel salicyl alcohol glycosyltransferase (UGT71L1). Over-expression of UGT71L1 in transgenic *Populus* led to a ~2-fold increase in concentrations of salicin and salicortin in roots. The quantitative effect on secondary metabolism is significant, as PGs are the second most abundant non-structural phenolics, after CTs, in the roots of this poplar clone. UGT71L1 is phylogenetically distinct from the well-characterized SAGTs. It belongs to a *Populus*-specific clade with no close homologs in *Arabidopsis*, *Vitis*,*Medicago* and *Oryza*, consistent with the limited taxonomic range of PGs. Overall, the experiments described highlight the important contributions of sugar transport and phenolic glycosylation to PG homeostasis, while also showing that SA and PGs make independent but complementary contributions toward the maintenance of *Populus* fitness.
